# Rare case of retropubic parasymphyseal cyst in a male patient

**DOI:** 10.1002/iju5.12382

**Published:** 2021-10-07

**Authors:** Shigeki Arase, Kiminobu Arima, Tomoki Kusafuka, Yasumitsu Kanamori, Hiroyuki Katou, Hiroshi Imai, Takashi Koyama, Takahiro Inoue

**Affiliations:** ^1^ Departments of Urology Kinan Hospital Minamimurogun Mihamacho Mie Japan; ^2^ Departments of Surgery Kinan Hospital Minamimurogun Mihamacho Mie Japan; ^3^ Pathology Division Mie University Tsu Mie Japan; ^4^ Department of Radiology Center and Diagnostic Radiology Kurashiki Central Hospital Kurashiki Okayama Japan; ^5^ Department of Nephro‐Urologic Surgery and Andrology Mie University Tsu Mie Japan

**Keywords:** antibacterial agents, magnetic resonance imaging, osteophyte, pubic symphysis, urinary bladder

## Abstract

**Introduction:**

Retropubic parasymphyseal cysta are rare, and few cases have been reported in men.

**Case presentation:**

A 65‐year‐old male patient presented with a 6‐month history of pelvic and perineal pain. Magnetic resonance imaging revealed a high‐intensity, irregular‐shaped mass extending from the pubic symphysis to the bladder. Contrast enhancement revealed no uptake in the central part of the mass, indicating a cystic component. Computed tomography showed erosion of the pubic symphysis and pubic osteophytes. Pathological findings of biopsy specimens revealed inflammatory fibrous tissue but no malignancy. The definitive diagnosis was retropubic parasymphyseal cyst associated with inflammation. The patient was treated with cefazolin from 1 day before surgery until postsurgical day 7. Oral antibiotic therapy was then prescribed for 1 month to maximize treatment. After 2 months, the patient’s symptoms resolved.

**Conclusion:**

Retropubic parasymphyseal cysts with inflammation and smaller asymptomatic cysts can be managed effectively with conservative or minimally invasive treatment.

**Abbreviations**
ALP: alkaline phosphatase
CRP: C‐reactive protein
CT: computed tomography
MRI: magnetic resonance imaging

**Keynote message**

Retropubic parasymphyseal cyst is a rare lesion that can cause severe symptoms. Careful evaluation is warranted to differentiate these lesions from malignant tumors and abscesses. A multidisciplinary team, including urologists, pathologists, and radiologists, is crucial for diagnosing and treating atypical cases such as this one. Retropubic parasymphyseal cysts with inflammation, as well as smaller asymptomatic cysts, may be managed effectively with conservative or minimally invasive treatment. After 2 months of antibiotic treatment, our patient’s symptoms resolved, and serum C‐reactive protein and alkaline phosphatase levels were normalized.

## Introduction

Retropubic parasymphyseal cysts are rare, and few cases have been reported in men. Retropubic parasymphyseal cysts may be asymptomatic or cause symptoms, such as urinary tract manifestations and pelvic pain. Because of the location, these cysts may be confused with malignant tumors and abscesses. In women, these lesions are thought to be caused by postmenopausal reactive changes as a result of multiparity.^1^ Sometimes, these cysts cause severe pain or are large enough to obstruct the urinary tract, in which case, invasive surgery may be necessary. Retropubic parasymphyseal cysts ≤3 cm in size in men shrink spontaneously after several years.^2^, ^3^ We describe the case of a man with a 5.4‐cm retropubic parasymphyseal cyst associated with inflammation.

## Case presentation

A 65‐year‐old man presented with a 6‐month history of lower abdominal, pelvic, and perineal pain. His medical history was unremarkable. He had previously been evaluated for prostate disease by a urologist, but no abnormalities were found.

**Fig. 1:**
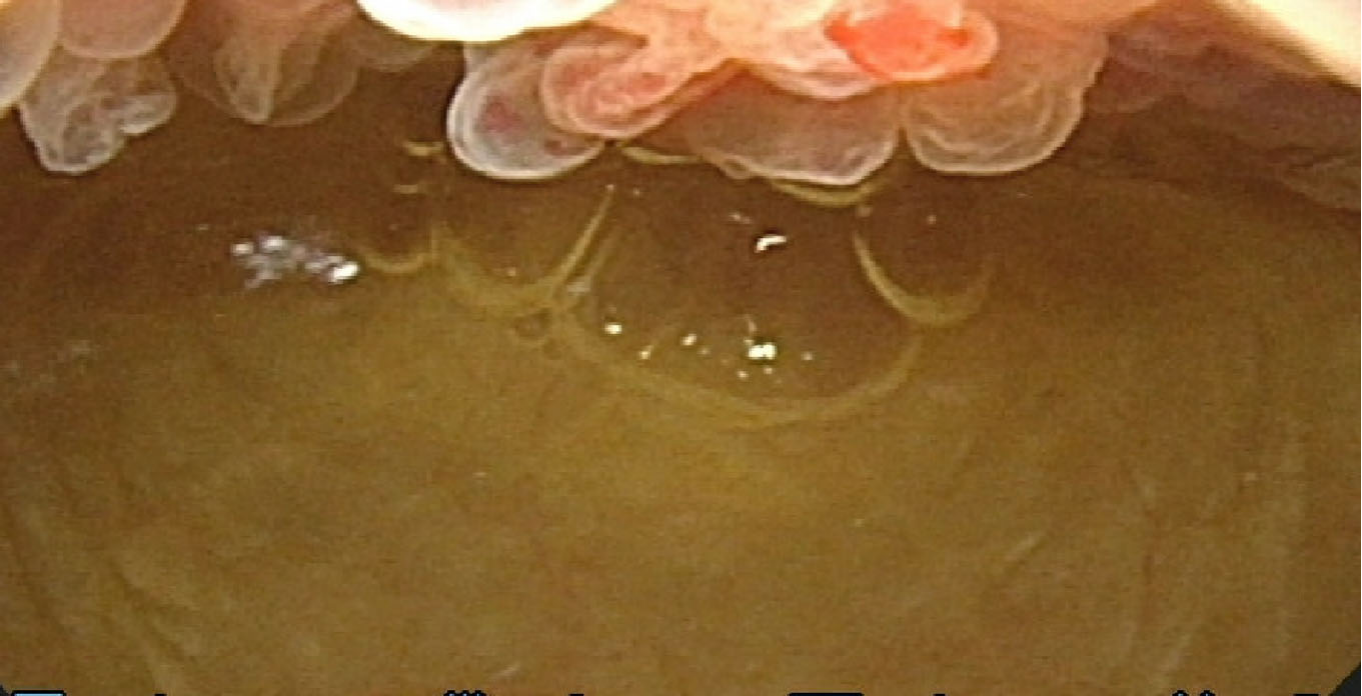
Cystoscopy demonstrating the edematous regions in the anterior of the bladder wall.

**Fig. 2:**
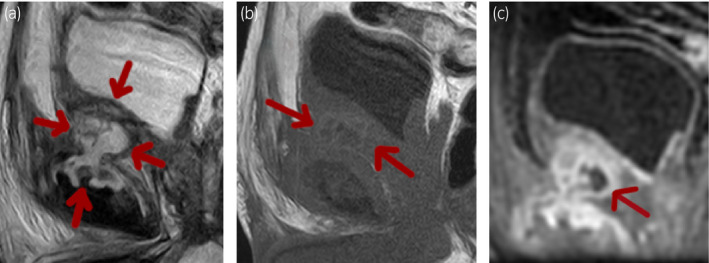
Fig. Magnetic resonance images. (a) Sagittal T2‐weighted image showing a high intensity, irregular‐shaped mass with a maximum diameter of 5.4 cm, extending from the posterosuperior aspect of the pubic symphysis to the anteroinferior aspect of the bladder (red arrows). (b) T1‐weighted image showing a mass of the same low intensity as the bladder wall. Notably, the central part of the mass exhibited a decreased signal (red arrows). (c) Contrast‐enhanced fat‐suppressed T1‐weighted image showing enhancement in the majority of the mass, except the center, which corresponded to the area of decreased intensity on unenhanced T1‐weighted imaging, thereby indicating a cystic component (red arrow).

**Fig. 3:**
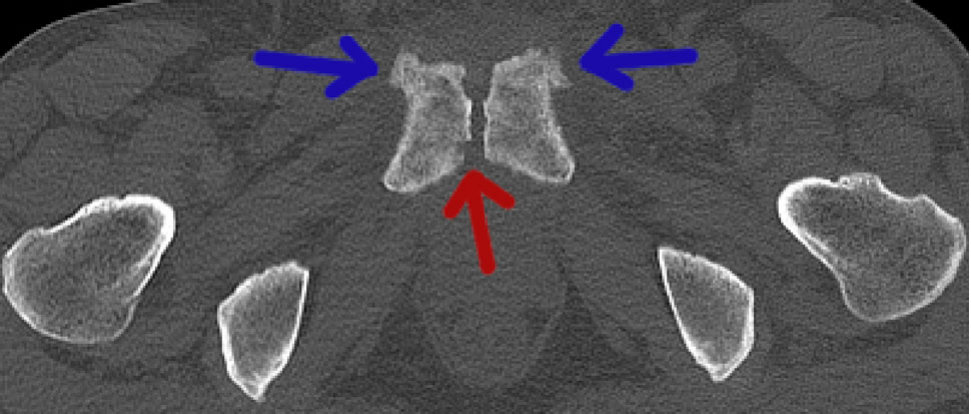
Computed tomography showing degenerative changes accompanied by erosion of the pubic symphysis (red arrow) and pubic osteophytes (blue arrows).

At admission, his urinalysis yielded normal findings; however, ultrasonography revealed thickening of the bladder wall. Cystoscopy demonstrated edematous regions in the anterior wall of the bladder (Fig. 1). Sagittal T2‐weighted MRI showed a high‐intensity, irregular‐shaped mass with a maximum diameter of 5.4 cm that extended from the posterosuperior aspect of the pubic symphysis to the anteroinferior aspect of the bladder (Fig. 2a). T1‐weighted imaging revealed a mass of similarly low intensity as the bladder wall. However, the central part of the mass exhibited decreased signal (Fig. 2b). On fat‐suppressed T1‐weighted imaging, gadolinium contrast material revealed enhancement in the majority of the mass. However, the central part of the mass showed decreased intensity on unenhanced T1‐weighted imaging and lacked contrast enhancement, thereby suggesting a cystic component (Fig. 2c). CT showed degenerative changes accompanied by erosion of the pubic symphysis and pubic osteophytes (Fig. 3). His serum tumor marker levels were normal; however, serum CRP was 4.25 mg/dL (normal range: 0.0–0.3) and ALP was 745 U/L (normal range: 120–340).

After case review, the multidisciplinary team recommended drainage of the atypical cyst and pathological examination to rule out malignancy. The pelvic pain hindered the patient’s mobility; thus, prompt diagnosis and treatment were necessary. He therefore underwent exploratory laparotomy.

We reached the pelvic cavity in laparoscopic procedure. No abscess was observed in the pelvic cavity; however, a cyst with inflammatory, hard fibrous tissue was observed around the pubic symphysis. We collected tissue specimens using forceps and placed a drainage tube over the cyst as laparoscopic fenestration. Only a small amount of serous drainage flowed out, and the drainage tube was removed after a few days.

**Fig. 4:**
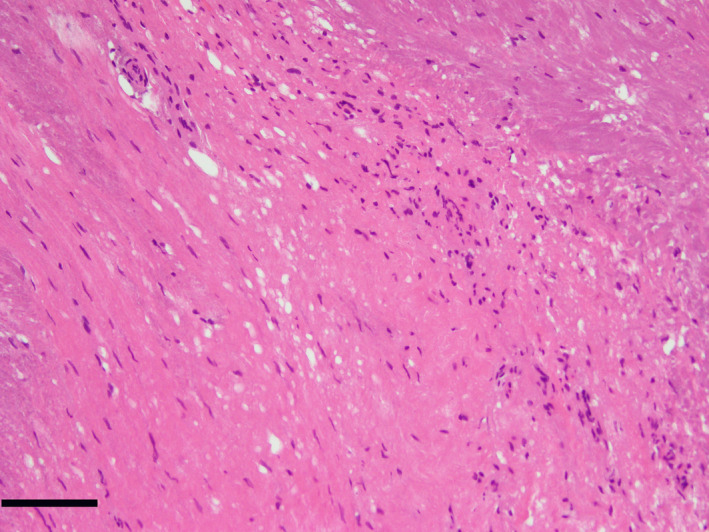
Pathological findings of the biopsy sample of the lesion revealed inflammatory fibrous tissue with lymphocytes and no malignancy. Dermal infiltrate; hematoxylin and eosin stain ×200 (Scale bar: 100 μm).

A biopsy sample of the lesion was obtained, and pathological findings revealed inflammatory fibrous tissue with lymphocytes but no malignancy (Fig. 4). The culture of the biopsy also yields negative results. The comprehensive diagnosis was retropubic parasymphyseal cyst associated with inflammation.

The patient was treated with cefazolin sodium 1 g IV q8h starting 1 day before surgery and continuing to postsurgical day 7. The patient’s symptoms rapidly improved, with serum CRP and ALP levels improving significantly. We continued antibiotic therapy minocycline 100 mg PO q12h for 1 month for optimal results. Over the next 2 months, the patient’s symptoms resolved, and serum CRP and ALP levels normalized.

## Discussion and analysis

Retropubic parasymphyseal cysts, are rare lesions that develop in the pubic symphysis, were first reported in 1996.^1^ The pubic symphysis is a hemiarthrotic joint comprising a fibrocartilaginous interpubic disc sandwiched between thin layers of hyaline cartilage.^4^ It is commonly affected by inflammatory arthropathy, infection, trauma, and degenerative changes.^5^ The typical clinical symptoms of retropubic parasymphyseal cysts include painful or painless vulvar mass, abdominal pain, urinary dysfunction, pain in the base of the penis, and sexual dysfunction.^6^ Signal characteristics on MRI are hypointense relative to muscle on T1‐weighted sequences, heterogeneously hyperintense on T2‐weighted sequences, and with a thin enhancing wall with no internal enhancement.^6^ Pathological findings of biopsy sampling or surgical resection are fibrocartilage or hyaline cartilage.^7^

In a literature review of 18 patients with retropubic parasymphyseal cysts, including 2 men and 16 multiparous women, Taniguchi et al.^7^ reported that the cyst size ranged from 1.5 to 7.2 cm (Table 1). Of the 16 women, 6 had a painless mass, whereas the others reported vulvar pain, urinary symptoms, difficulty with micturition, and acute urinary retention.^1^, ^6^, ^7^, ^8^, ^9^, ^10^, ^11^, ^12^, ^13^ Retropubic parasymphyseal cysts sometimes had to be distinguished from malignant tumors on ultrasonography‐guided needle biopsy, CT‐guided aspiration of the cyst, or open biopsy.^8^, ^9^, ^10^ Despite the benign results (fibrous connective tissue), surgical resection was necessary because of the size of the lesions and urinary tract symptoms.^1^, ^7^, ^8^, ^12^, ^13^

**Table. 1:** Clinical data on retropubic parasymphyseal cysts reported in the literature

Author, year	Age	Sex	Symptoms	Size	Treatment	Follow‐up result of observation
Alguacil‐Garcia et al., 1996^1^	58	Female	Pain and urinary dysfunction	40–50 mm	Operation	No recurrence over 1 year
	61	Female	Groin pain and paresthesia	20 mm	Operation	No recurrence over 3 years
Kim et al., 2004^8^	70	Female	Painless lump	40 × 40 mm	Operation	N/A
Martel et al., 2007^3^	72	Male	No symptom	30 mm	Observation	Size reduction after 6 months
Ergun et al., 2008^9^	54	Female	Chronic abdominal pain	N/A	Observation after needle biopsy	No change over 2 years
Gadde et al., 2011^11^	60	Female	Painless lump	46 mm	Cyst aspiration under CT guidance	N/A
Tan et al., 2012^10^	69	Female	Painless lump	50 × 30 × 30 mm 30 × 20 × 30 mm	Observation after open biopsy	N/A
Farag et al., 2014^12^	61	Female	Urinary dysfunction	32 × 30 × 39 mm	Operation	No recurrence over 2 months
	56	Female	Urinary dysfunction	30 × 38 × 27 mm	Operation	No recurrence over 4 months
Wylie et al., 2014^2^	69	Male	Pain in the base of penis and scrotum	25 mm	Observation	Size reduction after 4 years
Nishisho et al., 2016^6^	59	Female	Painful vulvar mass	15 × 10 × 10 mm	Observation	Mass resorption after 2 years
Strother et al., 2016^13^	62	Female	Painful vulvar mass	30 × 24 × 32 mm	Operation	N/A
Taniguchi et al., 2018^7^	62	Female	Lower abdominal pain and urinary retention	38 × 38 × 72 mm	Operation	No recurrence over 4 years
Present case	65	Male	Lower abdominal, pelvic, and perineal pain	54 mm	Biopsy under laparoscopic procedure and antibiotic therapy	Symptoms resolved after 2 months

In multiparous women, retropubic parasymphyseal cysts are thought to result from postmenopausal reactive changes.^1^, ^9^ In men, however, several reports indicated that cysts ≤3 cm in size shrink spontaneously after several years.^2^, ^3^ In their case reports, Wylie et al.^2^ suggested that in retropubic parasymphyseal cyst cases involving pain in the base of the penis and scrotum, the cyst reduction might have led to improvement in symptoms after 4 years. Conversely, in a patient for whom an asymptomatic retropubic parasymphyseal cyst shrank after 6 months, Martel and Spouge^3^ hypothesized that gas within the parasymphyseal cystic mass developed from a vacuum phenomenon in which gas patterns occurred transiently by negative pressure in the degenerated disc. In contrast, complete spontaneous regression was reported in a woman with a 1.5‐cm subpubic cartilaginous cyst.^6^ The authors speculated that the smallness of the cyst might have contributed to spontaneous resolution. The mass in our patient, in contrast, was large and associated with inflammation. Moreover, most retropubic parasymphyseal cysts are round, whereas the mass in our patient was irregular in shape, and the cyst was in the center of the mass.

We did not perform needle biopsy because the mass could not be identified by ultrasonography, and CT‐guided aspiration from an anatomically difficult location may cause tumor contamination, as reported previously.^6^ Consequently, we were able to obtain information on the mass in laparoscopy. The enhancement of most of the mass surrounding the cyst indicated subacute inflammation and spread to the bladder. This case was associated with inflammation and involved a larger cyst size than the previous cases of spontaneous contraction. We prescribed antibiotic therapy according to the general treatment for osteomyelitis^14^, ^15^ and laparoscopic fenestration. The patient was treated with intravenous cefazolin sodium during the perioperative period and placed on minocycline for 1 month for optimal treatment. Rifampicin was not used in combination due to side effects.

Exploratory laparotomy enabled fenestration of the mass, and antibiotic treatment was effective, as evident from the lack of symptoms at the 2‐month follow‐up. Communication among the multidisciplinary team, which included urologists, pathologists, and radiologists, was crucial for diagnosis and treatment in this atypical case.

## Conclusion

In rare retropubic parasymphyseal cyst cases, invasive surgery might be necessary when symptoms are severe, but conservative treatment or minimally invasive therapy may be effective and should be considered, as in this case report.

## Conflict of interest

The authors declare no conflict of interest.

## Approval of the research protocol by an institutional reviewer board

Not applicable.

## Informed consent

We obtained consent from our patient for the publication of this case.

## Registry and the registration no. of the study/trial

Not applicable.
